# Utilising clinical settings to identify and respond to the social determinants of health of individuals with type 2 diabetes—A review of the literature

**DOI:** 10.1111/hsc.12932

**Published:** 2019-12-18

**Authors:** Amanda Frier, Sue Devine, Fiona Barnett, Trisha Dunning

**Affiliations:** ^1^ College of Public Health, Medical and Veterinary Sciences James Cook University Townsville QLD Australia; ^2^ College of Healthcare Sciences James Cook University Townsville QLD Australia; ^3^ Faculty of Health Centre for Quality and Patient Safety Research Deakin University and Barwon Health Partnership Geelong VIC Australia

**Keywords:** clinical settings, literature review, social conditions, social determinants of health, social need, socio‐economic factors, type 2 diabetes

## Abstract

Type 2 diabetes (T2DM) is increasing in global prevalence. It is more common among people with poor social determinants of health (SDoH). Social determinants of health are typically considered at a population and community level; however, identifying and addressing the barriers related to SDoH at an individual and clinical level, could improve the self‐management of T2DM. This literature review aimed to explore the methods and strategies used in clinical settings to identify and address the SDoH in individuals with T2DM. A systematic search of peer‐reviewed literature using the electronic databases MEDLINE, CINAHL, Scopus and Informit was conducted between April and May 2017. Literature published between 2002 and 2017 was considered. Search results (*n* = 1,119) were screened by title and abstract against the inclusion and exclusion criteria and *n* = 56 were retained for full text screening. Nine studies met the inclusion criteria. Review and synthesis of the literature revealed written and phone surveys were the most commonly used strategy to identify social determinant‐related barriers to self‐management. Commonly known SDoH such as; income, employment, education, housing and social support were incorporated into the SDoH assessments. Limited strategies to address the identified social needs were revealed, however community health workers within the clinical team were the primary providers of social support. The review highlights the importance of identifying current and individually relevant social determinant‐related issues, and whether they are perceived as barriers to T2DM self‐management. Identifying self‐management barriers related to SDoH, and addressing these issues in clinical settings, could enable a more targeted intervention based on individually identified social need. Future research should investigate more specific ways to incorporate SDoH into the clinical management of T2DM.

**What is known about this topic**

- Social issues directly influence health, and are called social determinants of health (SDoH).
- Type 2 diabetes (T2DM) is more common among people with poor SDoH.
- SDoH are usually considered at a population level, not individually or clinically.

**What this paper adds**

- This is the first known literature review on how SDoH are incorporated into the clinical management of T2DM.
- Identified SDoH should be individually relevant, and considered a barrier to T2DM self‐management by the person with T2DM.
- There is a gap in formal methods and strategies to incorporate SDoH into usual clinical care for people with T2DM.

## 1 BACKGROUND

Diabetes prevalence has increased globally over the past three decades, with type 2 diabetes (T2DM) accounting for 85%–90% of all diagnoses (Diabetes Australia, 2015; World Health Organisation [WHO], 2016). People at socio‐economic disadvantage are more likely to develop T2DM and are more susceptible to suboptimal self‐management due to the consequences of poor social determinants of health (SDoH) (Australian Institute of Health & Welfare [AIHW], 2014, 2016). This socially influenced health disparity suggests a need to investigate strategies to optimise healthcare provision so that social disadvantage and SDoH are acknowledged and incorporated into the standard practice of T2DM care.

Social determinants of health are described as ‘the societal conditions in which people are born, grow, live, work and age’ (WHO, 2003). More specifically they include; early childhood development, education, employment, food security, housing, economic status, social support and healthcare access (Centres for Disease Control & Prevention [CDC], 2013; WHO, 2003). Social determinants influence both good and poor health. If a person is born into an affluent society with quality education, positive life circumstances, opportunity and healthcare access, the likelihood of good health is increased. To the contrary, when a person's lifespan is permeated with poor education, low economic status, unemployment, inadequate housing and limited access to quality healthcare, it is probable that their health status will be of poor quality, and they will have a shorter life expectancy (WHO, 2003).

Sustainable change towards improved SDoH requires political and social influence (Marmot & Wilkinson, 2006). Essential advocacy and action are underway at population and community levels (Keleher & MacDougall, 2016; Marmot & Wilkinson, 2006; Solar & Irwin, 2010); however while the approaches to address the causes of poor SDoH are occurring, the immediate and individual needs of people who live in circumstances contrary to a healthy life also require attention.

Despite the increasing prevalence of T2DM, especially amongst those at social disadvantage with poor SDoH (AIHW, 2014, 2016; Diabetes Australia, 2015; WHO, 2016), there are currently no published guidelines on how to consider T2DM and SDoH simultaneously, particularly at a clinical level. Living with suboptimal SDoH impedes the lifestyle choices essential for effective T2DM self‐management (Royal Australian College of General Practitioners [RACGP], 2016). Therefore, including strategies that identify and account for SDoH‐related barriers may augment usual care by allowing additional interventions to be instigated as part of standard clinical practice. This may be an additional step towards improving health outcomes for people with T2DM.

Health services could embed SDoH as part of standard practice. Identifying SDoH‐related barriers to T2DM self‐management could provide health professionals with insight into their clients’ life circumstances. Understanding an individual's SDoH and the associated health disparities could then help health professionals to develop more contextualised interventions (Baum et al., 2013; Newman, Baum, Javanparast, O'Rourke, & Carlon, 2015). The limited guidance to enable such an approach is stemmed from an overall deficit of supportive policies, frameworks and structure (Baum et al., 2013). This may also explain the lack of guidelines to incorporate SDoH into the clinical management of T2DM.

Although considering non‐medical issues is not the main focus in clinical settings, the relationship between poor SDoH and the ability to self manage diabetes is supported by an extensive evidence base (Brown et al., 2004; Kumari, Head, & Marmot, 2004; Marmot, 2005; WHO, 2003). Therefore the formal incorporation of SDoH into usual clinical management of T2DM deserves more in‐depth consideration and strategic progression.

Incorporating SDoH into T2DM clinical care; by identifying, considering and subsequently addressing the related self‐management barriers could improve T2DM outcomes by enabling the ability to make the positive lifestyle choices required for effective T2DM self‐management. This in turn, could help reduce the personal suffering that often accompanies the burden of living with diabetes.

## 2 METHODS

### 2.1 Aim of the review

This literature review aimed to explore methods and strategies used in clinical settings to identify and address the SDoH of individuals with T2DM. It is worth noting the word ‘address’ and its synonyms should not be interpreted as resolving the SDoH issue. Instead, the correct interpretation is the strategies used to accommodate for the identified SDoH issue. For example, if it had been identified that a patient has limited transport options which would therefore impact their healthcare access, then arranging appropriate transport could alleviate the consequences of these SDoH issues.

The initial focus on identifying individuals’ SDoH‐related issues was to gain insight into what factors were included, and how and when SDoH identification could be incorporated into routine T2DM clinical care. Strategies and recommendations to address the identified SDoH issues were then explored to determine how the related barriers to T2DM self‐management could be addressed.

### 2.2 Systematic approach

The varied methodologies used in the reviewed studies (Table 3) indicated the suitability of an integrative approach to the literature review (Whittemore & Knafl, 2005), however it's iterative and interpretive nature is similar to that of a scoping review (Arksey & O'Malley, 2005). Consequently the current review borrowed from both styles of literature review. Both follow a systematic process which includes;

1. research question formulation
2. systematic literature searching
3. study selection (informed by inclusion and exclusion criteria)
4. quality appraisal
5. analysis and interpretation
6. summarising, collating and reporting.

(Arksey & O'Malley, 2005; Whittemore & Knafl, 2005).

### 2.3 Search strategy

The PRISMA protocol (Liberati et al., 2009) for searching literature guided a systematic search of the computerised databases MEDLINE, CINAHL, Scopus and Informit. Keywords, synonyms and associated truncations, including MeSH terms, were categorised into three groups; SDoH, T2DM and clinical setting (Table 1).

**Table 1:** Categorised groups of keywords, synonyms and truncations

Group	SDoH	T2DM	Clinical Setting
Synonyms & truncations	Health social determinants Social determinants of health Social determinants Socioeconomic Socioeconomic factors Socio‐economic factors Socioeconomic status Health status disparity Health status disparities Health disparity Health disparities Social conditions Social circumstances Societal conditions Societal circumstances Societal factors SES	Adult onset diabetes Ketosis resistant diabetes MODY Maturity onset diabetes Maturity‐onset diabetes NIDDM Non‐insulin dependent diabetes Noninsulin dependent diabetes Slow‐onset diabetes Slow onset diabetes Stable diabetes Type 2 diabetes Type ii diabetes	Primary care clinic Health service Health services Community healthcare providers Health centre Health centres Health clinic Health clinics Health care providers Community health workers community health worker Clinic setting Family medicine Medical care Medical centre Health workers Health worker Healthcare providers Healthcare provider Health personnel Clini*

The search was limited to papers published between 2002 and 2017 English language and human studies. The 15‐year search scope was applied to identify publications influenced by ‘Social Determinants of Health: The Solid Facts (second edition)’ (WHO, 2003). This publication was considered important because it preceded an increasing evidence base concerning the influence of social determinants on health. The keywords were combined to obtain the primary search results.

Titles and abstracts were screened to ensure all of the included articles discussed clinical settings, identification and/or addressed the SDoH‐related issues of individuals with T2DM. Incorporating the keywords (or their synonyms) identification* and/or address* into the search strategy appeared to eliminate pertinent articles, thus manual screening of titles and abstracts was necessary. After the initial screening and duplicate removal, the full text of the articles were read in brief. The inclusion and exclusion criteria were then applied to the remaining articles (Table 2).

**Table 2:** Inclusion and exclusion criteria

Inclusion criteria	Exclusion criteria
≥18 years	<18 years
SDoH and T2DM in clinical settings AND	Type 1 diabetes
Identifying* SDoH of individuals (strategies to identify/screen/assess/measure) AND/OR	Gestational diabetes
Addressing* SDoH (recommendations only to include) AND/OR	Acute settings
Addressing* SDoH (practical strategies to address)	Area/region identification* of SDoH issues rather than on an individual level
Published in a peer‐reviewed journal	Policy/upstream approaches to addressing* SDoH (only) rather than on an individual level

**Figure 1:**
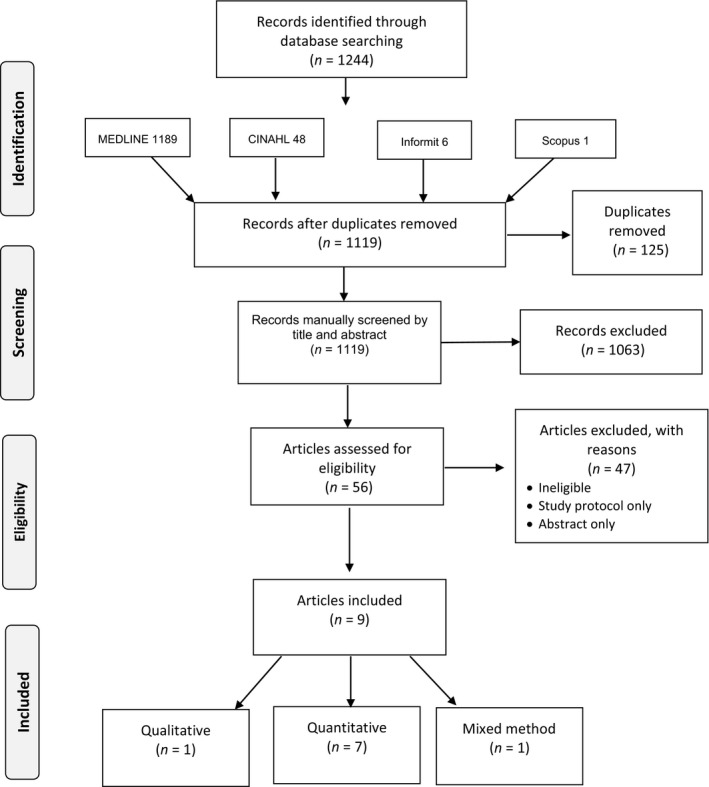
PRISMA flowchart of article identification, screening, eligibility and inclusion

The search identified 1,244 articles. One hundred and twenty‐five duplicates were removed, leaving 1,119 articles. Title, abstract and text screening reduced the remaining articles to 56. The inclusion and exclusion criteria were applied to these 56 articles. Nine articles remained and were included in the review. Figure 1 outlines the process followed to identify, screen for eligibility and to include and exclude articles.

### 2.4 Critical review, data extraction and analysis

Each study was critically reviewed using the McMasters critical appraisal tools for both quantitative and qualitative studies depending on the methodology used (Law et al., 1998; Letts et al., 2007). One study used mixed methods; therefore, both quantitative and qualitative McMasters appraisals were conducted for that study (Loh, Jaye, Dovey, Lloyd, & Rowe, 2015). The reviewed studies were then summarised and collated for comparison and interpretive analysis (Table 3). Commonly known SDoH (WHO, 2003) provided a reference for determining which SDoH were identified, and how frequently they were included (Table 4). The methods and strategies used to elicit this information were also ascertained during the study reviews (Table 5).

**Table 3:** Summary of articles reviewed

Citation	Title abbreviation	Study goal	Study design	Study methodology	Setting and sample	Study findings	Methods and strategies used to identify & address SDoH issues
Gimpel et al. (2010)	Patient perceptions of a community‐based care coordination system.	To assess the efficacy of including CHW as care coordinators into education programs/groups to address social concerns, and provide clinical support to patients with T2DM and depression	Exploratory	Focus groups	Community‐based setting. Dallas USA ‘Project Access Dallas‐care coordination system’ *N* = 24	Participants reported the support of community‐based workers as a helpful inclusion. Benefits were also reported in participating in groups e.g. social support and understanding	***Identifying*** Modified risk assessment tool (identifying‐social concerns, risk of developing T2DM, depression) ***Addressing*** Include strategies to address SDoH, for example, how use public transportation and facilitating access to healthcare. Incorporated the use of community health workers
Walker et al. (2014a)	Independent effects of socioeconomic and psychological social determinants of health on self‐care and outcomes in T2DM	To investigate independent effects of socio‐economic and psychological SDoH factors on DM knowledge, self‐care and QoL	Cross‐sectional	Statistical analyses to provide information on individual and collective contribution of different SDoH to T2DM	Adult primary care clinic USA *N* = 615	*T2DM knowledge and self‐care:* Significantly associated with SES and psychological components of SDoH *T2DM outcomes*: Significantly associated with higher SES and self‐efficacy and lower diabetes distress and perceived stress *QoL:* Significantly associated with higher education, lower depression, lower psychological distress, lower perceived stress, and higher social support	***Identifying*** Participants completed validated questionnaires ***Addressing*** Not Included Recommendations for further research to inform future interventions designed to improve self‐care and outcomes for patients with T2DM
Walker et al.. (2014b)	Relationship between SDoH and processes and outcomes in adults with T2DM: validation of a conceptual framework	To validate a conceptual framework that clarifies the pathways linking SDoH to health outcomes of people with T2DM.	Cross‐sectional	Path analysis used to determine if SDoH factors independently predict glycaemic control, or show an association with mediators/moderators of T2DM care components	Adult primary care clinic USA. *N* = 615	Significant paths were associated with SDoH and glycaemic control through direct association and mediators/moderators of diabetes care components	***Identifying*** Participants completed validated questionnaires ***Addressing*** Recommendation to include SDoH in future research and T2DM intervention
Walker et al. (2015a)	Quantifying Direct Effects of SDoH on Glycemic Control in Adults with T2DM	To investigate if self‐care is the pathway through which SDoH impact T2DM outcomes	Cross‐sectional	Structured equation modelling investigated the relationship between SDoH, self‐care and glycaemic control	Adult primary care clinic USA *N* = 615	An association between self‐care and SDoH is suggested, but is not mediated by self‐care A direct relationship identified between psychosocial determinants of health and glycaemic control	***Identifying*** Participants completed validated questionnaires ***Addressing*** Interventions should take psychosocial factors into account as independent influences on T2DM outcomes, rather than influences on self‐care
Walker et al. (2015b)	Understanding the influence of psychological and socioeconomic factors on DM self‐care using structured equation modelling	To develop and test latent variables of SDoH that influence diabetes self‐care	Cross‐sectional	Confirmatory factor analysis identified the latent factors underlying socio‐economic determinants, psychosocial determinants and self‐care Structured equation modelling was used to investigate the relationships between the above determinants and self‐care	Adult primary care clinic USA *N* = 615 Self‐efficacy, psychosocial distress and social support also had an influence over behaviour	Psychosocial factors can be separated into three latent constructs; psychological distress, social support and self‐efficacy Better self‐care is associated with lower psychological distress, higher social support and higher self‐efficacy	***Identifying*** Participants completed validated questionnaires ***Addressing*** Consider psychosocial, self‐efficacy, social support and psychological distress separately rather than collectively Incorporate behavioural and psychological strategies in future T2DM interventions
Walker et al. (2015)	SDoH in adults with T2DM‐Contribution of mutable and immutable factors	To increase understanding about the role of multiple SDoH factors on glycaemic control of individuals with T2DM To identify which SDoH factors are, mutable and immutable	Cross‐sectional	Statistical analysis using a hierarchical model with HbA1c as a dependent variable with block independent variables i.e. Demographics, socio‐economic, psychosocial, built environment, clinical, and knowledge/self‐care	Adult primary care clinic USA *N* = 615	Significant associations with HbA1c included self‐efficacy, social support, comorbidity, insulin use, medication adherence and smoking behaviour SDoH factors that drive glycaemic control are modifiable and therefore worthy of inclusion in health interventions	***Identifying*** Participants completed validated questionnaires ***Addressing*** Recommendations for greater acknowledgement of SDoH required to reduce the commodities associated with glycaemic control. Recommendations for DM education and skills training to include SDoH factors
Loh et al. (2015)	Dunedin's free clinic: an exploration of its model of care using case study methodology	To determine if the services provided met the social vulnerability need of clients	Mixed method Descriptive (nested case study)	Created a profile of patient need using various measures. Then applied an analytic matching technique to assess the degree of alignment between services provided and patient need	Community‐based free health clinic NZ *N* = 406 *nested case study* *n* = 21 *medical certificates n = 278* *justice use n = 80* *surveys completed n = 27*	Patient need complicated by coexisting social vulnerability. Suggested a degree of fit between the services provided and the need of the patients. Highlighted importance of a model of care that caters for patients with complex social need	***Identifying*** Collected patient need through journal entries, patient encounters, self‐administered surveys, medical certificates issued, hospital admissions, justice system use, and computer database records ***Addressing*** Not Included
Rose (2005)	Socioeconomic Barriers to DM Self‐care: Development of a Factor Analytic Scale	To describe the development of a measurement tool for assessing SES barriers to T2DM self‐care	Cross‐sectional Part of a mixed method study investigating socio‐cognitive factors/barriers accompanying DM self‐care (quantitative component)	Theoretical constructs followed by telephone surveys to develop SES assessment measures Factor analysis on SES‐related diabetes self‐care barriers	Diabetes register from Fairfield division of GP’s. Australia *N* = 105	SES barriers identified through the factor analysis consists of ‘place barriers’ and ‘information barriers’ SES cost‐related barriers failed to form one factor in the analysis Further development required	***Identifying*** Phone survey developed using theoretical constructs ***Addressing*** Not Included
Rosland et al. (2014)	Social Support and Lifestyle versus. Medical DM Self‐Management in the Diabetes Study of Northern California (DISTANCE)	To examine the relationship between social support and T2DM self‐management/lifestyle behaviours, and self‐management/medical behaviours	Cross‐sectional	Self‐management and social support, including SDoH factors assessed using the DISTANCE questionnaire, and administrative data Poisson regression models to estimate ARR of self‐management behaviours at high and low levels of social support	Integrated managed‐care consortium. California, USA *N* = 13,366	Clearer association with high levels of self‐support and positive self‐management/lifestyle behaviours compared to medical behaviours	***Identifying*** DISTANCE survey specifically designed to assess self‐management behaviours of T2DM patients. Includes social support and SDoH factors ***Addressing*** Not included

**Table 4:** SDoH factors included in the reviewed studies

SDoH factor	Included in screening
Access to medical/healthcare	9/9 studies
Income	8/9 studies
Education	7/9 studies
Employment	7/9 studies
Social support	7/9 studies
Subjective social status (social gradient)	6/9 studies
Psychological or emotional distress (stress)	6/9 studies
Financial constraints	3/9 studies
Transport	3/9 studies
Health literacy	2/9 studies
Food security	1/9 studies
Housing	1/9 studies
Social exclusion	1/9 studies
Early life	1/9 studies

**Table 5:** Summary of methods used to identify SDoH issues

Study title	Citation	Methods used to conduct SDoH screening
Patient perceptions of a community‐based care coordination system	Gimpel et al. (2010)	Modified risk assessment tool (survey). The survey was designed to identify social concern and need. Also provided a description of SES indicators in participant descriptions i.e. education, employment and income. No indication if survey was self‐administered or assisted
Independent effects of socioeconomic and psychological social determinants of health on self‐care and outcomes in T2DM	Walker et al. (2014a)	Numerous individual and validated assessment tools: Survey assessing household income, years of education and employment status Social Support Survey Subjective Social Status –pictorial ladder to indicate perceived social status. Perceived Stress Scale Short version of the Test of Functional Health Literacy in Adults Also provided a description of SES status indicators in participant descriptions i.e. education, employment and income. No indication if assessment tools were self‐administered or assisted
Relationship between SDoH and processes and outcomes in adults with T2DM: validation of a conceptual framework	Walker et al. (2014b)	Numerous individual assessment tools: Interview survey assessing household income, years of education and employment status Social Support Survey Subjective Social Status –pictorial ladder to indicate perceived social status. Perceived Stress Scale Short version of the Test of Functional Health Literacy in Adults Also provided a description of SES status indicators in participant descriptions i.e. education, employment and income No indication if assessment tools were self‐administered or assisted
Quantifying Direct Effects of SDoH on Glycemic Control in Adults with T2DM	Walker et al. (2015a)	Numerous individual assessment tools: Interview survey assessing household income, years of education and employment status Social Support Survey Subjective Social Status –pictorial ladder to indicate perceived social status. Perceived Stress Scale Short version of the Test of Functional Health Literacy in Adults Also provided a description of SES status indicators in participant descriptions i.e. education, employment and income No indication if assessment tools were self‐administered or assisted
Understanding the influence of psychological and socioeconomic factors on DM self‐care using structured equation modelling	Walker et al. (2015b)	Numerous individual assessment tools: Interview survey assessing household income, years of education and employment status Social Support Survey Subjective Social Status –pictorial ladder to indicate perceived social status. Perceived Stress Scale Short version of the Test of Functional Health Literacy in Adults Also provided a description of SES status indicators in participant descriptions i.e. education, employment and income No indication if assessment tools were self‐administered or assisted
SDoH in adults with T2DM‐Contribution of mutable and immutable factors	Walker et al. (2015)	Numerous individual assessment tools: Interview survey assessing household income, years of education and employment status Social support survey Subjective social status–pictorial ladder to indicate perceived social status. Perceived Stress Scale Short version of the test of functional health literacy in adults Also provided a description of SES status indicators in participant descriptions i.e. education, employment and income No indication if assessment tools were self‐administered or assisted
Dunedin's free clinic: an exploration of its model of care using case study methodology	Loh et al. (2015)	Retrospective data collection via journal entries, patient encounters, medical certificates, patient medical records and databases. Also provided a description of SES indicators in participant descriptions i.e. unemployment, sickness benefits, and accommodation
Socioeconomic Barriers to DM Self‐care: Development of a Factor Analytic Scale	Rose (2005)	Phone surveys based on items that indicate SES barriers to T2DM self‐care i.e. cost/finances, transport, food security, safety and health literacy
Social Support and Lifestyle versus. Medical DM Self‐Management in the Diabetes Study of Northern California (DISTANCE)	Rosland et al. (2014)	Self‐administered/report questionnaire. Included comprehensive SDoH assessment i.e. access to medical/healthcare, income, education, employment, social support, social gradient, stress, financial constraints, transport, health literacy, food security, housing, social exclusion, early life. Also included many other T2DM management‐related components. 185 questions in total

## 3 RESULTS

### 3.1 General characteristics of studies

Seven of the nine studies included in the review were quantitative, one was qualitative and one used a mixed method design. Four articles were published in 2015. Three were published in 2014 and one in 2010 and 2005 respectively. The age of participants in the reviewed studies ranged from 30–75 years. Sample sizes for eight of the studies ranged from *n* = 24 to *n* = 615. The remaining study was extremely large at *n* = 13,366. Seven of the studies were completed in the United States (USA), one in New Zealand and one in Australia.

Only one study intentionally investigated the value of identifying and addressing the SDoH‐related issues of individuals with T2DM in a clinical setting. The remaining studies did not purposefully investigate identifying and/or addressing SDoH‐related needs; however their methodology indirectly included these factors. Five of the nine articles were written by the same authors using the same data set. Each article reported separate interactions and relationships between T2DM and SDoH using different statistical analyses to investigate the specific issues considered in each study. Each study was published individually, and met the inclusion criteria for the current review. Consequently these five studies were appraised individually. All studies included a description of their ethics or approval procedures. Table 3 provides an overview of the articles included in the review.

### 3.2 Identification of SDoH‐related issues

#### 3.2.1 What was included?

Although identifying SDoH issues was not the primary focus for most of the reviewed studies, all embedded SDoH screening into their study protocol. Identification of social need was conducted as part of the study design or within participant descriptions, or both. Overall, SDoH factors included; income, employment, access to medical/healthcare, education, health literacy, social support, social exclusion, subjective social status (social gradient), serious psychological distress (stress), financial constraints, transport, food security, housing and early life. Table 4 displays the identified SDoH factors, and the number of studies that included them in their screening process.

#### 3.2.2 When and how was it done?

All studies completed the SDoH assessment prior to commencing the research protocol. Various approaches were used to gather the desired information. These were: written surveys (self‐administered and assisted), phone surveys, health clinic databases and records, and medical chart entries. Table 5 provides a summary of the strategies and methods used to assess the SDoH‐related issues of individuals.

### 3.3 Addressing SDoH‐related issues

Only one of the nine studies included specific strategies to address the identified SDoH‐related needs of people with T2DM (Gimpel et al., 2010). The provided support was guided by the participant's identified social need obtained in the initial SDoH assessment. Community health workers undertook a care coordination/case management role which involved assisting study participants to navigate the healthcare system independently. Examples of CHW assistance included arranging translation services, home visits, appointment reminders, supporting health education strategies, and teaching participants how to use public transport. Enrolment in the program also involved cost reduction of consultations and medications for participants. This strategy addressed financial constraints and issues associated with low income (Gimpel et al., 2010).

Walker et al.’s five studies (2014a, 2014b, 2015a, 2015b, 2015) demonstrated multiple interactions and relationships between T2DM and SDoH. Consequently, they recommended SDoH be incorporated into T2DM management and interventions. Their recommendation did not provide any insight into how to address SDoH issues. However, the authors did recommend further research be conducted to inform and improve self‐care and outcomes for people with T2DM by incorporating SDoH‐based strategies (Walker et al., 2014a). Use of the same data set for these five studies is acknowledged and discussed in the limitation section of this review.

The remaining three studies acknowledged the relationship between SDoH and T2DM; however none of the studies provided any specific recommendations or strategies about how to incorporate SDoH in into T2DM care (Loh et al., 2015; Rose, 2005; Rosland et al., 2014).

## 4 DISCUSSION

The aim of this literature review was to explore the methods and strategies used in clinical settings to identify and address the SDoH of individuals with T2DM. Review of the approaches used to identify SDoH‐related issues revealed informative factors that could inform routine SDoH assessments in the clinical setting (Table 5). Although practical strategies to address the identified SDoH‐related barriers to T2DM self‐management were limited, the associated recommendations provided valuable insight to inform future intervention and research.

### 4.1 Identifying social need

Social determinants of health mean that the social factors in a person's life determine their health status and outcomes (Marmot & Wilkinson, 2006). The interdependent relationship between SDoH, T2DM and health outcomes was clear in Walker et al.’s five articles (2014a, 2014b, 2015a, 2015b, 2015). The SDoH factors they included were: income, education, subjective social status, serious psychological distress, access to healthcare and social support. These closely align with the key SDoH factors described by leading health organisations (AIHW, 2016; CDC, 2013; WHO, 2011).

Although Walker et al. (2014a, 2014b, 2015a, 2015b, 2015) demonstrated an unequivocal interdependence between T2DM and SDoH, they did not indicate whether the participants regarded the SDoH‐related issues as barriers to effective T2DM self‐management. In contrast, Gimpel et al. (2010) used focus groups to evaluate the effectiveness of CHWs employed to screen and address the social and economic concerns of individuals with, or at risk of T2DM and depression. Their SDoH screen was completed using a modified health risk assessment survey (Table 5). The findings indicated the primary concerns of participants were: condition specific and self‐management education, financial constraints, effective communication, respect, access to medication and transport. The qualitative nature of data collection enabled participants to share their personal experiences about how poor SDoH and social vulnerability affected their self‐management of T2DM.

Social vulnerability information was collected retrospectively by Loh et al. (2015) (Table 5). Identifying SDoH‐related issues in a retrospective manner, such as reviewing medical records and patient encounter data, as done by Loh et al., possibly negates articulation of current barriers to T2DM self‐management, and may reflect the researchers’ interpretation of SDoH‐related barriers, rather than the actual barriers encountered by the person with T2DM. Focusing on perceived barriers to T2DM self‐management would enable personal insights based on lived experience and current circumstances to be explored and documented (Liamputtong, 2013).

Rosland et al. (2014) asked about current situations and perceived barriers to self‐management using a self‐administered survey. This survey specifically assessed the perspectives of people with diabetes (Kaiser Permanente', 2005), and is part of a longitudinal study in Northern California (Kaiser Permanente', 2017; Moffet et al., 2009). The long but comprehensive survey (185 questions) incorporated: income, employment, education level, health literacy, transport, healthcare access, social gradient, social support, social exclusion, emotional distress, early life, housing and food security. Using personal perspectives on well‐known SDoH could bring greater meaning and relevance to identifying SDoH‐related barriers to the self‐management of T2DM.

Rose (2005) also assessed patient views about barriers to T2DM self‐management. The study was undertaken to inform the development of a tool to measure the socio‐economic barriers for people with diabetes. Participants in the study completed a phone survey, which used a five‐point Likert scale to assess socio‐economic barriers to diabetes self‐management. The findings were inconclusive with sample size inaccuracy identified as a possible cause. Nonetheless, the author stressed the need to investigate the socio‐economic impact on diabetes outcomes, and discussed the importance of continued progression on a reliable and valid measure of socio‐economic barriers to diabetes self‐care (Rose, 2005).

Employment and income were two of the most frequently assessed SDoH (7/9 and 9/9 respectively). These SDoH constituents are interrelated, because employment status can affect level of income, and insufficient income can increase financial constraints. The three studies that included financial constraints (Gimpel et al., 2010; Rose, 2005; Rosland et al., 2014) incorporated the consequences of personal income status, which provided some insight into how this SDoH factor can be a barrier to T2DM self‐management.

Lack of income and financial constraints also limit healthcare access when people cannot afford adequate healthcare (Keleher & MacDougall, 2016; WHO, 2003). Limited access to healthcare is a known barrier to achieving good health (WHO, 2011). All of the reviewed studies included access to medical/healthcare, which highlights the importance of asking people about their healthcare access, and prioritising it in an SDoH assessment.

Ability to access health services is also limited by a lack of transport (Keleher & MacDougall, 2016; New South Wales Council of Social Service [NCOSS], 2012). This association is widely acknowledged throughout the literature (AIHW, 2016; WHO, 2011, 2003). Rosland et al. (2014) qualified this by including questions on how transport deficits contribute to reduced healthcare access. Despite the well‐defined relationship between transport and healthcare access, only three studies included transport in their SDoH screening (Table 4).

Insufficient transport, employment and income can also exacerbate social exclusion as a lack of these can inhibit people's ability to access social networks (Keleher & MacDougall, 2016). Seven of the nine reviewed studies incorporated social support, and Rosland et al. (2014) also included social exclusion. The interaction between social support, social exclusion and T2DM management was evidenced in Strom and Egede’s (2012) systematic literature review. They concluded that higher levels of social support contributed to positive T2DM outcomes and the associated lifestyle behaviours.

Healthy lifestyle behaviours are integral to optimal T2DM self‐management (Egger, Binns, & Rossner, 2011; RACGP, 2016). In addition, effective diabetes self‐management depends on adequate health literacy, which is augmented by quality education (Kim, 2016; Kim & Lee, 2016). Education is a widely recognised SDoH factor (AIHW, 2016; CDC, 2013; WHO, 2011): accordingly, seven of the nine reviewed studies included education when assessing an individual's SDoH.

Rosland et al. (2014) and Rose (2005) combined education and health literacy with individual perspectives by considering the reading ability and comprehension of their study participants. This suggests that screening for health literacy, in place of educational attainment may be a more informative inclusion in an SDoH assessment. Wallace, Carlson, Malone, Joyner, and Dewalt (2010) and Welch, Van Geest, and Caskey (2011) advocated for health literacy rather than education level, to be incorporated into patient screening. Their use of health literacy assessment tools negated interpretation of education quality and level, and allowed for a more current and relevant assessment to be completed. Of note, the authors did acknowledge the limitations of health literacy screening tools (Wallace et al., 2010; Welch et al., 2011).

Interestingly, despite the importance of considering health literacy, the reviewed studies appeared to provide minimal assistance to help participants complete SDoH screens. Rose (2005) conducted phone interviews, which would have enabled provision of verbal explanations when needed. The remaining studies relied on written responses which could increase the likelihood of systematic measurement error (Büettner & Muller, 2011), and contribute to inaccurate responses.

Social positioning is a well‐established SDoH (AIHW, 2016; CDC, 2013; WHO, 2011). Walker et al. (2014a, 2014b, 2015a, 2015b, 2015) and Rosland et al. (2014) used an assessment tool to measure social positioning. This SDoH assessment item was subjective, and asked individuals’ to indicate their perceived position within society. It was not specified how this perception extended to T2DM self‐management; however, social positioning has a well‐known relationship with health status (Marmot, 2003; WHO, 2003) and renders it deserving of more in‐depth investigation into the value of including it in an SDoH assessment.

Food security, housing, addiction and early life are also well recognised SDoH (AIHW, 2016; CDC, 2013; Marmot, 2003; WHO, 2011, 2003), as is their relationship with the self‐management of T2DM (WHO, 2003; Yu & Raphael, 2004). Rosland et al. (2014) were the only authors to consider these SDoH factors. However because of their well‐known association to health, their inclusion in an SDoH assessment requires also further exploration.

Stress is arguably one of the most critical aspects to consider when identifying an individuals SDoH (Marmot & Wilkinson, 2006; WHO, 2003)‐related barriers to T2DM self‐management. It can occur as a ‘result of social and psychological circumstances’ (WHO, 2003). The studies by Walker et al. (2014a, 2014b, 2015a, 2015b, 2015) and Rosland et al. (2014) incorporated stress in their SDoH assessment. They measured it in individually relevant terms; however the perceived impact of stress on T2DM self‐management could not be interpreted.

Stress is increased with the coexistence of insufficient income, unemployment, social exclusion, inadequate transport, poor housing and food insecurity. This harmful accumulation of SDoH factors leads to people feeling they lack control over their lives (Keleher & MacDougall, 2016; WHO, 2003); in turn, this affects T2DM self‐management (Brown et al., 2004; WHO, 2003; Yu & Raphael, 2004).

The evident multifactorial and interconnected nature of SDoH confirms that no single SDoH constituent works in isolation (Brown et al., 2004). Consequently, the convoluted and expansive impact of the SDoH combined with their apparent effect on T2DM self‐management should be considered collectively when identifying SDoH‐related barriers in the context of diabetes self‐care.

### 4.2 Addressing the identified social need

Very few tangible strategies for addressing the identified SDoH‐related issues were identified. Individual SDoH circumstances and whether they were perceived as barriers to T2DM self‐management appear to be central to how and what should be addressed. In addition, targeted and formalised integration of SDoH into clinical care through collaboration and partnerships between health services, community supports and social services is required (Baum et al., 2013; Freeman, Javanparast, Baum, Ziersch, & Mackean, 2018; Newman et al., 2015). Though this provides an informative starting point, further work in the area is needed, including the development of guidelines and policies (Baum et al., 2013).

Community health workers in Gimpel et al.'s study (2010) provided support based on the patient's perception of the identified SDoH issues as barriers to T2DM self‐management. In addition to providing condition specific education, the CHWs developed individualised patient care plans and provided support such as; referrals to social and healthcare services, assistance with medication and screening, transport assistance, translation services, health education, home visits, appointment reminders, and supported links to other community services. Gimple et al. (2010) also suggested group‐based interventions could be helpful, and have a role in empowering participants by improving T2DM knowledge, self‐management capacity and providing condition‐specific social support.

Social support was identified by Rosland et al. (2014) as being linked with lifestyle‐related self‐management behaviours. The authors acknowledge the worthiness of future investigation into the provision of social support to improve diabetes self‐management. Appointing CHWs to focus on enhancing social support could help address SDoH‐related barriers to T2DM self‐management. This notion is supported by J. Freeman (2016) and McCalmont et al. (2016) who advocate for CHWs to work as part of the clinical team to address SDoH‐related issues.

It is also noteworthy that participation in the program discussed by Gimpel et al. (2010) included a cost reduction of medications and treatment services. This is an important inclusion, as it addresses barriers associated with limited income and financial constraints. This strategy was depicted as an enabler to T2DM self‐management by study participants.

Though not specific to T2DM, momentum towards addressing SDoH in clinical settings has commenced in Canada and the USA (Andermann, 2013, 2016, 2018; Page‐Reeves et al., 2016). In particular, the ‘Community Links Evidence to Action Research’ (CLEAR) collaboration incorporates SDoH factors in the toolkit they have developed. The CLEAR collaboration toolkit provides general direction on SDoH screening domains in clinical settings. It also outlines a ‘patient level, practice level and community level’ approach to addressing identified social issues (Andermann, 2013). Health professionals who have used the toolkit indicate that it provides contextualised guidance about how to screen for and address the SDoH‐related issues of vulnerable patients in clinical settings (Naz, Rosenberg, Andersson, Labonté, & Andermann, 2016). The toolkit was not specifically developed for T2DM, therefore determining its applicability and clinical relevance is required before extrapolating it into diabetes care.

Combining the ‘CLEAR toolkit’ approach with including CHWs as part of the clinical team, to specifically address SDoH issues, may enhance the recommendations provided by the CLEAR collaboration (Andermann, 2013). Benefits similar to this were identified by Hunt, Grant, and Appel (2011). Their review of 16 articles found obvious benefits of incorporating CHWs into T2DM management because of their capacity to work at patient, health professional, health clinic and community levels. A broad interpretation of CHW was applied in the Hunt et al. (2011) article by using the term community health advisor (CHA). Their definition of a CHA included CHWs, peer and various health, and diabetes support workers. The CHA’s provided transport, support for appointments and emotional issues, various social support activities, and assistance with literacy and comprehension (Hunt et al., 2011). The authors concluded that CHA’s services are highly effective and valued by both participants and healthcare providers. Similar assistance was described in the reviewed study by Gimpel et al. (2010). The value of including CHW/CHA input to address SDoH‐related issues for individuals with T2DM and in clinical settings appears persuasive and is well supported (Andermann, 2016; Gimpel et al., 2010; Hunt et al., 2011; Naz et al., 2016).

Supporting client literacy and comprehension is an integral role of a CHW/CHA (Gimpel et al., 2010; Hunt et al., 2011). People with lower levels of education are accurately presumed to have worse health literacy (Keleher & MacDougall, 2016; Kim, 2016; Wallace et al., 2010). The ‘inability for individuals to access, understand, appraise and communicate health information within the healthcare system and the wider community’ (Keleher & MacDougall, 2016) contributes to reduced healthcare access, suboptimal self‐management (Welch et al., 2011) and contributes to a cascade of poor health outcomes resulting from poor SDoH. Poor health literacy leads to an inability to optimise diabetes education and support services, and therefore can lead to a deficit in diabetes knowledge and understanding. In turn, this can affect an individual's ability to achieve optimal T2DM self‐management (Bains & Egede, 2011; Schillinger, Barton, Karter, Wang, & Adler, 2006). The quality of diabetes care is therefore dependent on a health professional's ability to accommodate for client health literacy levels (Wallace et al., 2010).

The benefit of including diabetes education that is sensitive to health literacy is supported by Kim and Lee (2016). Their systematic review and meta‐analysis of 13 relevant articles focused on strategies to accommodate for patients with low health literacy. They found an overall improvement in glycaemic management when health literacy was addressed. This provides convincing support for the integration of health literacy into diabetes self‐management interventions (Kim & Lee, 2016; Wallace et al., 2010).

## 5 LIMITATIONS

The term ‘social determinants of health’ was only defined in the MEDLINE electronic database at the beginning of 2014, although it entered mainstream literature in approximately 2003. Prior to 2014 the phrases socio‐economic status, socio‐economic factors and social conditions were used. To overcome this, a variety of synonyms were used in the search strategy; however it is possible some relevant literature may have been missed.

Including the terms ‘identifying’ and ‘addressing’ (and their synonyms) in the electronic database search inaccurately narrowed the search results to zero, and subsequently they were not used. Similarly an unmanageable amount of literature was produced when the synonyms of health equity, equality, inequity and inequality were included. Consequently manual screening of titles and abstracts was necessary prior to applying the inclusion and exclusion criteria. This may have limited the search, and is therefore worthy of acknowledgement.

Use of the same data set in the five articles by Walker et al. (2014a, 2014b, 2015a, 2015b, 2015) limited the breadth of the current literature review by reducing the total number of approaches used to identify the SDoH of individuals with T2DM in clinical settings. Although SDoH were only identified once, each study used different statistical analyses to describe separate interactions between SDoH and T2DM, and thus all were included in the review.

Expanding the search to include other chronic diseases such as heart disease and stroke may have yielded more results, as the influence of SDoH on these conditions is also acknowledged (WHO, 2003), however this would have detracted from the specific focus on T2DM. Furthermore, this limitation also sheds light on the paucity of research currently done on SDoH in clinical settings, where T2DM is usually managed.

## 6 CONCLUSION

Social determinants of health and T2DM are interdependent, and inadequate self‐management of T2DM is more common in those with poor SDoH (AIHW, 2014, 2016). Consequently the benefit of considering SDoH in conjunction with T2DM self‐management was evident in the literature. The aim of the literature review was to explore methods and strategies used in clinical settings to identify and address the SDoH of individuals with T2DM. The literature did not reveal any specific guidelines; however, synthesis of the reviewed studies and associated literature revealed informative direction for future research.

Identifying social need in a clinical setting requires an individualised approach. Considering the individuals’ personal circumstances and whether they perceive the SDoH‐related issue as a barrier to T2DM self‐management brings relevance to well‐recognised SDoH. Thereby incorporating an individualised approach to assess SDoH‐related barriers to T2DM self‐management into clinical settings could enable a more targeted approach to usual clinical care.

Considering health literacy rather than education level may enhance the usability and application of SDoH assessments by allowing for improved comprehension of the terminology frequently used in T2DM care. Furthermore, accommodating for health literacy is crucial when identifying SDoH‐related barriers, and when addressing SDoH‐related issues. This combined with the expertise and skills of CHWs may be advantageous when devising strategies to incorporate SDoH into the clinical management of T2DM.

The impetus towards including SDoH in clinical settings has begun in Canada and the USA (Andermann, 2013, 2016; Page‐Reeves et al., 2016), and the strategies outlined in the CLEAR toolkit (Andermann, 2013) could be contextualised and then incorporated into the clinical management of T2DM.

Current efforts to advance T2DM management could be enhanced by incorporating innovative approaches that include the SDoH as part of standard clinical practice. Contextualising and progressing current approaches used in clinical settings to identify and address SDoH‐related barriers to T2DM self‐management could enable this approach. Furthermore, it is an opportunity to expand strategies that address SDoH and contribute to improved health equity in general.

## CONFLICT OF INTEREST

All authors declare that there are no conflicts of interest.

## AUTHOR'S CONTRIBUTIONS

The review was led by AF. SD and FB provided methodological guidance including design, search strategy, appraisal and synthesis of the reviewed articles. Literature searching was conducted by AF. This included searching databases, importing records, removing duplicates and record screening. Appraisal of article quality, synthesis and interpretation of findings was conducted by AF with final results confirmed by SD and FB. AF led the writing of the review. SD, FB and TD provided guidance on the overall content and structure of the literature review. SD, FB and TD were responsible for critically revising the literature review. All authors (AF, SD, FB and TD) read and approved the final manuscript.
